# Clinico-hemato-biochemical profile of dogs with liver cirrhosis

**DOI:** 10.14202/vetworld.2015.487-491

**Published:** 2015-04-12

**Authors:** M. A. Elhiblu, K. Dua, J. Mohindroo, S. K. Mahajan, N. K. Sood, P. S. Dhaliwal

**Affiliations:** 1 Department of Veterinary Medicine, Guru Angad Dev Veterinary and Animal Sciences University, Ludhiana - 141 004, Punjab, India; 2 Department of Veterinary Surgery and Radiology, Guru Angad Dev Veterinary and Animal Sciences University, Ludhiana - 141 004, Punjab, India; 3 Department of Teaching Veterinary Clinical Complex, Guru Angad Dev Veterinary and Animal Sciences University, Ludhiana - 141 004, Punjab, India

**Keywords:** biochemistry, coagulation profile, dogs, hematology, liver cirrhosis, ultrasonography

## Abstract

**Aim:**

The aim of this study was to determine the relevant tools in the diagnosis of liver cirrhosis in dogs.

**Material and Methods:**

A total of 140 dogs presented at Veterinary Teaching Hospital, Guru Angad Dev Veterinary and Animal Sciences University, Ludhiana, showing clinical signs of hepatic insufficiency were subjected to clinico-hemato biochemical, urological, ultrasonographic (USG), and USG guided fine-needle biopsy examinations by standard methods. On the basis of these results, 6 dogs out of 140 dogs were found to be suffering from liver cirrhosis. Six clinically healthy dogs constituted the control group.

**Results:**

The dogs suffering from liver cirrhosis manifested inappetence, halitosis, abdominal distension, weight loss, melena, icterus, anemia, and neutrophilic leukocytosis with the left shift. Levels of hemoglobin, lymphocytes, packed cell volume, mean corpuscular volume, mean corpuscular Hb (MCH), and platelet count were significantly lower in liver cirrhosis group than control group while total leukocyte count, neutrophils, and MCH concentration were significantly higher. Glucose, total protein, albumin, A/G ratio, and fibrinogen were significantly lower, and creatinine, alanine aminotransferase, aspartate aminotransferase, alkaline phosphatase, prothrombin time, and APTT were significantly higher than the control values. Ultrasound revealed diffuse increase in echogenicity with rounded and irregular liver margins. Cytological examination of the ascitic fluid and fine-needle aspiration biopsy of liver was not fruitful in the diagnosis of liver cirrhosis.

**Conclusions:**

Liver cirrhosis causes clinical and hemo-biochemical alterations, which require special consideration when treating diseased animals. USG, diffuse increase in echogenicity of liver, rounding and irregularity of liver margins and microhepatica were the consistent findings. It is suggested that USG along with hemo-biochemical alterations may be used as a diagnostic tool for liver cirrhosis in dogs.

## Introduction

Chronic hepatitis is recognized and well-documented liver disorder in canines [1]. Cirrhosis is the end-stage of chronic hepatitis and is defined as a diffuse distribution characterized by fibrosis of the liver and the conversion of normal liver architecture into structurally abnormal nodules, micro- or macro-nodules [2]. It is considered irreversible, although the point at which this happens is not well-defined [2]. Although chronic hepatitis is a regularly diagnosed condition in dogs, liver cirrhosis is less frequently encountered [3]. Until now, no antifibrotic therapy has been clinically available in dogs, and in humans, liver transplantation remains the only treatment option in cases of hepatic dysfunction resulting from cirrhosis.

Although hemato-biochemistry is considered as important preliminary tools for proceeding toward the correct diagnosis and treatment protocol, but radiography is useful to evaluate the morphologic abnormalities and ultrasonography (USG) is an excellent non-invasive way to evaluate liver parenchyma [4]. The literature about hematological and biochemical alteration in liver cirrhosis is scarce.

As per our knowledge, there were no previous published reports on clinical finding and laboratory alterations of liver cirrhosis under Indian conditions. Hence, the prospective present study was undertaken to investigate several aspects of blood biochemical profile in dogs with liver cirrhosis.

## Materials and Methods

### Ethical approval

The study was conducted after the approval of the Institutional Animal Ethics Committee. All owners gave consent for dogs to be included in the study and undergo the testing procedures.

### Study population

The present study was conducted on 140 dogs of both genders, aged 6 months to 14 years and body weight ranged from 2.9 kg to 51 kg. The dogs were presented at Small Animal Clinics of Teaching Veterinary Hospital, Guru Angad Dev Veterinary and Animal Sciences University (GADVASU), Ludhiana, India, with signs of inappetence, polyuria (PU), polydipsia (PD), abdominal distension, weight loss, anemia, and jaundice. All 140 dogs were diagnosed to be suffering from hepatic insufficiency on the basis of clinico-hemato-biochemical and USG findings. The six dogs that were finally included in the study (from a total of 140 initially referred) fit the definition of liver cirrhosis.

### Signalment and anamnesis

Data collected when the animals were examined included breed, age, sex, and time (days) from the onset of clinical signs. History of feed intake, water intake, fecal color, symptoms of pain, and any prior treatment given were noted in every case.

### Clinical examination

Each animal was subjected to a detailed clinical examination. Each animal was thoroughly evaluated for its general condition, inspection of mucous membranes, hydration status, signs of pain, and abdominal distension.

### Hematology

Blood samples (2 ml) were collected aseptically from the cephalic vein in ethylenediaminetetraacetic acid coated vials (Accuvote-PLUS, Quantum Biologicals Pvt.Ltd. Chennai, Tamil Nadu, India). Immediately after collection, the blood was used for determination of hemoglobin (Hb), packed cell volume (PCV), total leukocyte (TLC) count, total erythrocyte count (TEC), erythrocyte indices- mean corpuscular volume (MCV), mean corpuscular Hb (MCH), MCH concentration (MCHC), platelet count, and differential leukocyte count by and automated hematology analyzer (ADVIVA 2120 Hematology System, Siemens). Further, a thorough examination of a stained blood smear was also done to determine any left shift and toxic changes in neutrophils.

### Clinical biochemistry

For serum biochemical analysis, blood samples were collected in serum vials. After clotting, serum was separated by centrifugation and transferred to a dry clean vial for further evaluation. For glucose and coagulation profile estimation, blood samples were collected in sodium fluoride and sodium citrate coated vials (Accuvote Disposables), respectively. VITROS DT60 II chemistry system (Ortho-Clinical Diagnostics, Johnson and Johnson Company, New Brunswick, NJ, USA) was used to determine the serum activities of alanine aminotransferase (ALT), aspartate aminotransferase (AST), alkaline phosphatase (ALP), gamma-glutamyltransferase (GGT), total bilirubin, total proteins, albumin, blood urea nitrogen (BUN), creatinine, cholesterol, and plasma glucose. Plasma fibrinogen was estimated by heat precipitation method using a hand-held refractometer. Prothrombin time (PT) and activated partial thromboplastin time APTT were assessed by using commercially available reagents of TULIP DIAGNOSTICS (P) LTD. (Goa, India).

### Radiography and USG

All the six dogs were subjected to chest and abdominal X-ray, abdominal USG, and ultrasound guided fine needle aspiration biopsy (USG-FNAB) using 23G needle, by standard procedures.

### Other ancillary tests

Abdominocentesis was performed in all six dogs by standard protocol and under aseptic condition. Abdominocentesis revealed clear ascitic fluid in all six cases and was subjected to cytological examination. Urine samples were subjected to physical and laboratory examination. The urine samples were centrifuged and the urine sediment was examined microscopically.

### Statistical analysis

All quantitative data were presented as mean ± standard error. The comparison between control and diseased group was done using unpaired t-test. Significance was set at p<0.05 and p<0.01.

## Results

### Clinical observations

Liver cirrhosis was diagnosed in six cases out of the total 140 cases of hepatic insufficiency presented to our clinics based on clinical examination, laboratory evaluation, and USG findings. The animals suffering from liver cirrhosis were Labradors (4 male and 2 female) with age ranging from 5 to 12 years. The duration of the illness ranged from 5 to 20 days with a mean of 9.2±2.4 days. The clinical signs of dogs suffering from liver cirrhosis were inappetence, halitosis, melena, hematochezia, PU, PD, dehydration, icterus, weight loss, and abdominal distension.

### Hemo-biochemical and other laboratory findings

The mean of hematological and biochemical values of the control group and liver cirrhosis groups are presented in Table-1. The mean values of Hb (p<0.05), PCV (p<0.05), lymphocytes (p<0.01), MCV (p<0.01), MCH (p<0.01), and platelets (p<0.01) were significantly lower in liver cirrhosis group than control group, while TLC (p<0.01), neutrophils (p<0.01) and MCHC (p<0.01) were significantly higher than the control group. Anemia was observed in all the cases, thrombocytopenia in two and thrombocytosis in one case. Toxic changes in neutrophils were observed in four animals (mild to moderate in three and severe in one). Left shift was mild to moderate in four and marked in two animals.

**Table-1:** The hemato-biochemical changes in healthy and liver cirrhosis dogs on the day of the presentation.

Parameter	Control (*n*=6)	Liver cirrhosis (*n*=6)
Hb (g/dL)	12.22±0.29	9.68±4.5*
TEC (10^6^/µL)	5.59±0.10	4.46±0.52
TLC (10^3^/µL)	13.9±0.67	32.98±15.81*
N (%)	58.67±3.04	89.66±6.74**
L (%)	38.67±2.23	10.00±6.32**
E (%)	2.67±0.84	0.33±0.8
PCV (%)	39.93±0.82	24.54±4.87*
MCV (fL)	71.20±0.49	49.15±2.89**
MCH (pg)	21.70±0.32	19.32±0.08**
MCHC (g/dL)	30.47±0.24	39.94±2.16**
Platelets (10^5^/µL)	2.94+0.39	1.94±0.27**
GLU (mg/dL)	100.50±3.12	83.67±5.18*
BUN (mg/dL)	13.67±0.96	28.67±6.31
Creatinine (mg/dL)	0.92±0.04	1.60±0.16**
TP (g/dL)	6.37±0.29	4.78±0.37**
ALB (g/dL)	3.35±0.24	1.62±0.15**
Globulin (g/dL)	2.92±0.34	3.17±0.26
A/G ratio	1.26±0.21	0.52±0.04*
Total bilirubin (mg/dL)	0.37±0.09	1.90±0.87
ALT (U/L)	28.00±3.34	61.00±13*
AST (U/L)	36.67±3.91	57.83±17.46*
ALP (U/L)	52.50±9.67	376.50±54.71**
GGT (U/L)	2.50±0.50	9.67±0.57
Cholesterol (mg/dL)	176.67±24.99	124.83±28.53
PT (s)	6.9±0.09	10.9±1.1*
APTT (s)	12.4±1.3	18.3±1.6*
Fibrinogen (g/dL)	2.1±0.08	0.96±0.02*

SE=Standard error, Hb=Hemoglobin, TEC=Total erythrocyte count, PCV=Packed cell volume, MCV=Mean corpuscular volume, MCH=Mean corpuscular hemoglobin, MCHC=Mean corpuscular hemoglobin concentration, TLC=Total leukocyte count, N=Neutrophils, L=Lymphocytes, E=Eosinophils, GLU=Glucose, BUN=Blood urea nitrogen, TP=Total proteins, A/G ratio=Albumin:Globulin ratio, ALT=Alanine aminotransferase, AST=Aspartate aminotransferase, ALP=Alkaline phosphatase, GGT=Gamma-glutamyl transferase,

* Significance at 5% (p<0.05);

** Significance at 1% (p<0.01)

There was a significant increase in the concentration of ALT (p<0.05), AST (p<0.05), ALP (p<0.01), creatinine (p<0.01), PT (p<0.05), and APTT (p<0.05) in the liver cirrhosis group compared to the control group. The concentrations of glucose (p<0.05), total proteins (p<0.01), albumin (p<0.01), A/G ratio (p<0.05), and fibrinogen (p<0.05) were significantly lower in the liver cirrhosis group compared to the control group. Although BUN and GGT were much higher that the control group they did not differ significantly.

Cytological examination of the ascitic fluid and fine needle aspiration biopsy of the liver were not fruitful in the diagnosis of liver cirrhosis. Examination of urine sediment smear showed bilirubin casts in three cases with mild to moderate hyperbilirubinemia.

### Radiography and USG

**Figure-1:**
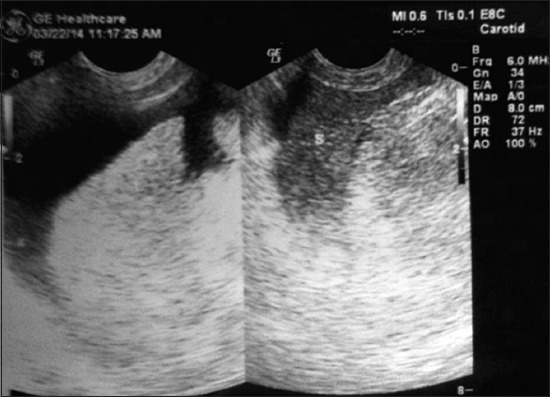
An ultrasonogram of cirrhotic liver shows generalized hyperechoic hepatic parenchyma with rounded and slightly irregular liver margins.

**Figure-2:**
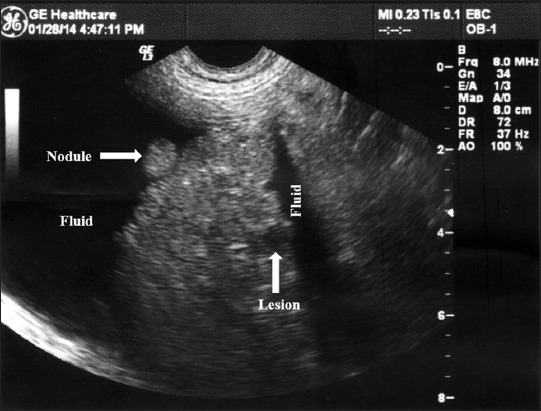
An ultrasonogram of cirrhotic liver shows generalized hyperechoic hepatic parenchyma with multiple hyperechoic lesions, irregular margins, and multiple small nodules on the surface.

**Figure-3:**
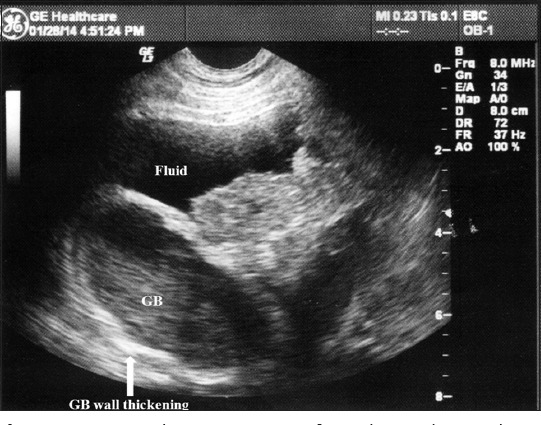
An ultrasonogram of cirrhotic liver shows distended gall bladder with the wall thickened and lots of free anechoic fluids in the abdominal cavity (ascites).

X-ray revealed no significant finding in any of the dogs examined. USG of liver revealed diffuse increase in echogenicity as compared to spleen, so-called “bright liver” in all the cases (Figure-1). In all the cases, there was rounding off the liver margins. Microhepatica, which is a common finding of hepatic cirrhosis was observed in five cases. In four of the cases, distension of gall bladder with thickened wall was observed (Figure-2). Mild hepatic congestion was seen in two cases, i.e., the hepatic veins were moderately dilated and visualized prominently in the hepatic parenchyma. In other two cases, there was an irregularity of liver margins (Figure-3). Multiple hypoechoic lesions with multiple small nodules on the surface of liver parenchyma were observed in one case (Figure-3).

## Discussion

To the authors’ knowledge, this is the first study under Indian conditions in which signalment, clinical signs, hemo-biochemical findings, diagnostic procedures, were investigated in a series of cases of liver cirrhosis. One of the aims of the study was to investigate the occurrence of liver cirrhosis. The prevalence of liver cirrhosis in the present study was 4.3%, which may be actually higher, owing to the inclusion of dogs with hepatic insufficiency only. It was not possible to establish the etiology during this study.

Melena and hematochezia observed has been ascribed to the gastrointestinal ulceration or coagulopathies, which may be due to hyperfibrinolysis, where the patients with advanced hepatocellular liver disease and cirrhosis produce decreased thrombin activatable fibrinolysis inhibitor [5]. The coagulation abnormalities were observed in this study and seem to be the most probable explanation for melena. Weight loss could be due to the inadequate nutrient intake as a result of inappetence and enhanced tissue catabolism [6] and abdominal distension was ascribed to ascites. The signs of icterus, PU, PD, and dehydration observed in this study have been earlier reported in hepatic disorders [1,7], but these signs have not been specifically documented for liver cirrhosis of dogs. Jaundice and icterus are hallmark of hepatic disorders [1] and clinically hepatic dysfunction may be manifested by signs of diarrhea, PU, PD and dehydration [7,8]. PU and PD have been attributed to impaired adrenal steroid metabolism, altered portal vein osmoreceptor, loss of renal medullary concentration gradient, and encephalopathy [6].

The anemia was attributed to chronic nature of this disease due to increased transient time of erythrocytes through the spleen due to reduced portal blood flow and/or fragility of red blood cells due to high levels of bile acids [9-11]. Since liver cirrhosis is a consequence of chronic hepatitis, neutrophilic leukocytosis and left shift indicated inflammatory response of chronic hepatitis. Several mechanisms have been suggested for thrombocytopenia in patients with liver disease, including increased platelet sequestration in the spleen as a result of congestive splenomegaly; reduced production of thrombopoietin by the liver; increased platelet breakdown due to auto-antibodies, and increased consumption resulting from low-grade disseminated intravascular coagulopathy (DIC) [5]. The significantly increased PT and APTT, and low platelet count and fibrinogen indicated DIC. Although DIC was suspected in all cases of this study, splenomegaly was not observed on USG. Similar to present findings, earlier researchers have reported anemia, leukocytosis, and thrombocytopenia in liver cirrhosis cases [9,12,13].

Although AST and ALT, weresignificantly higher that the control group, the mean values of these parameters were within the normal reference range for dogs. Normal ALT, AST, and GGT values could be due to fibrosis of most of the hepatocytes. Font *et al*. [13] also reported normal hepatic enzyme levels in liver cirrhosis. High ALP could indicate primary hepatic disease; however, in dogs ALP is not liver-specific and its elevation may be due extrahepatic sources [14]. Hence, high ALP levels, in this study may be of extrahepatic origin. Increased serum bilirubin could be due to the damage of hepatocytes and decreased elimination. Bilirubinuria observed in few cases was suggestive of underlying hepatic disease [11].

Hypoproteinemia is the most common finding in chronic disorders like cirrhosis and portosystemic vascular abnormalities [15] because liver is the main site for synthesis and degradation of the proteins. A low serum albumin concentration due to liver disease indicates a diffuse and chronic hepatopathies [16], and the same nature of the disease was observed in this study. Decreased nutrient uptake associated with hepatopathies may be another possible reason for hypoalbuminemia. Similar present observations, hypoproteinemia, and hypoalbuminemia have been reported by other workers, in chronic hepatic disorders [4,17,18]. Hypocholesterolemia may be due to long-lasting liver disease as a result of the drop in the production or absorption from the intestines or higher conversion to bile acids [19]. Renal dysfunction has been reported as a frequent complication in patients with end-stage liver disease [20]. Hence, the increased in BUN and creatinine values could be attributed to impaired kidneys function associated with liver cirrhosis due to the decreased capacity of the liver to detoxify the harmful products.

Microhepatica observed in the present study has been reported as a common finding of hepatic fibrosis or cirrhosis [4,17] and is due to replacement of parenchymal tissue by fibrous tissue. The USG findings of the present study are in concurrence with that of Biller *et al* [21].

## Conclusion

Liver cirrhosis was characterized by inappetence, halitosis, abdominal distension, weight loss, PU, PD, melena, and icterus. Levels of Hb, lymphocytes, PCV, MCV, MCH, platelet count, and fibrinogen were significantly lower in liver cirrhosis group than the control group while TLC, neutrophils, MCHC, PT, and APTT were significantly higher. Glucose, total proteins, albumin, A/G ratio, and fibrinogen were significantly lower, and creatinine, ALT, AST, ALP, PT and APTT were significantly higher than the control values. Cytological examination of the ascitic fluid and fine-needle aspiration biopsy of liver was not fruitful in the diagnosis of liver cirrhosis. USG, diffuse increase in echogenicity of liver, rounding and irregularity of liver margins and microhepatica were the consistent findings. It is suggested that USG along with hemato-biochemical alterations may be used as a diagnostic tool for liver cirrhosis in dogs. To conclude, liver cirrhosis causes clinical and hemo-biochemical alterations, which require special consideration when treating the diseased animals.

## Authors’ Contributions

The present study was part of MAE’s Ph.D. Dissertation. KD designed the study and approved the experimental protocol. MAE performed the experiment. MAE and KD drafted and revised the manuscript. JM and KSM helped in ultrasonography. NKS helped in cytological examination. PSD guided and helped in clinical evaluation of animals. All authors read and approved the final manuscript.
